# Risk of Hepatocellular Carcinoma Remains High in Patients with HBV-Related Decompensated Cirrhosis and Long-Term Antiviral Therapy

**DOI:** 10.1155/2020/8871024

**Published:** 2020-12-17

**Authors:** Dong-Mei Zhu, Jing Xie, Chun-Yan Ye, Mei-Yun Qian, Yuan Xue

**Affiliations:** ^1^Department of Liver Diseases, The Third People's Hospital of Changzhou, Changzhou, Jiangsu, China; ^2^Institute of Hepatology, The Third People's Hospital of Changzhou, Changzhou, Jiangsu, China

## Abstract

**Background:**

This study aimed to evaluate the risk factors of HCC development in patients with hepatitis B virus (HBV)-related DC and who underwent long-term antiviral therapy.

**Methods:**

Data from 308 patients with HBV-related DC and long-term antiviral therapy were collected and retrospectively reviewed. Cox regression analysis was used to analyze independent risk factors of HCC development.

**Results:**

Data from 129 patients with definite records were analyzed. The median follow-up time was 5 years (range, 1 to 8 years). At the end of the follow-up, 41 (31.8%) patients developed HCC, and the time from DC diagnosis to HCC incidence who received antiviral therapy was 4.4 years (range, 1–7 years). The incidence of HCC was higher in males (30/78, 38.5%) than in females (11/51, 21.6%) (*P* = 0.04). Patients who developed HCC were significantly older than those who did not develop HCC (*P* < 0.01). The incidence of HCC in patients receiving nucleoside analogues, nucleotide analogues, and combination therapy was 34.7%, 38.1%, and 33.3%, respectively, and the difference showed no significant differences (*P* = 0.95). Multivariate Cox regression analysis demonstrated that male gender and age ≥50 years are independent risk factors of HCC development (OR = 2.987 and 2.408; 95% CI (1.301–6.858) and (1.126–5.149); *P* = 0.01 and 0.02, respectively).

**Conclusion:**

The risk of HCC remains to be high in patients with HBV-related DC, especially in males aged ≥50 years.

## 1. Introduction

Hepatitis B virus (HBV)-related liver cirrhosis (LC) leads to portal hypertension, hepatic dysfunction, and subsequent decompensation in individuals. Due to serious complications such as ascites, esophagogastric variceal bleeding, hepatic encephalopathy, or hepatocellular carcinoma (HCC), patients with decompensated cirrhosis (DC) had higher mortality than those with compensated LC [1]. Oral antiviral drugs such as tenofovir disoproxil fumarate (TDF) and entecavir (ETV) suppress HBV replication, improve liver function, and reduce mortality [2]. Despite long-term antiviral therapy, the risk of HCC persists over time according to a multicenter study conducted in Korea [3]. During the 5-year follow-up, 9.0% patients have developed HCC, while cumulative incidence of HCC for Caucasians with chronic hepatitis B (CHB) was 2.4% at age 8. Age, baseline LC status, and liver stiffness were shown to be risk factors of HCC [4]. However, studies on the risk factors for the development of HCC in patients with DC are rare.

Moreover, increasing evidence has revealed that TDF is superior over ETV in reducing HCC in patients with chronic HBV infection. Data from a study conducted in Korea have shown that the cumulative incidence rates of HCC in patients treated with TDF were lower than those treated with ETV, suggesting that TDF treatment significantly lowered the risk of HCC when compared to ETV [5]. Subsequently, three meta-analysis studies have reported similar findings [6–8]. Nevertheless, in another study, TDF was reported to be inferior over ETV in reducing HCC development [9]. To our knowledge, the comparative effectiveness of nucleotide and nucleoside analogues in reducing the risk of HCC remains unclear in patients with DC.

We herein explored the cumulative incidence of HCC in patients with DC and long-term antiviral therapy through an 8-year follow-up and analyzed the risk factors associated with HCC development.

## 2. Patients and Methods

### 2.1. Patients

The data from 308 patients with HBV-related DC who were admitted to the Department of Liver Diseases, the Third People's Hospital of Changzhou from January 2011 to January 2016, and received long-term antiviral therapy were retrospectively reviewed. DC in patients was diagnosed according to the China's Guidelines for the Prevention and Treatment of Chronic Hepatitis B [10]: LC with at least one episode of ascites, jaundice, hepatic encephalopathy, or variceal bleeding. HCC was diagnosed based on imaging techniques, alpha-fetoprotein (AFP), tests, or biopsy [11]. Patients with malignant tumor and coinfection with other hepatitis virus were excluded from the study.

Demographic and clinical data, including age, gender, family history, clinical symptoms, and antiviral therapy, were collected. Laboratory data including hepatitis B e antigen (HBeAg) and HBV DNA, alanine aminotransferase (ALT), AFP, platelet counts, bilirubin, creatinine, and international standard ratio were collected at the time of admission.

All patients with DC underwent abdominal ultrasonography, AFP, and biochemical tests at an interval of 3 to 6 months during the follow-up period. Patients who developed HCC within 6 months were excluded. The study endpoint included HCC development, death, or lost to follow-up. After HCC diagnosis, the patients were transferred to the Department of Oncology or Hepatobiliary Surgery and then underwent surgical resection, radiofrequency ablation, transarterial chemoembolization, or combination therapy according to the Guidelines for Diagnosis and Treatment of Primary Liver Cancer in China (2019 edition) [11]. This was a noninterventional study, and the study protocol was approved by the Ethics Committee of the Third People's Hospital of Changzhou, according to the Declaration of Helsinki, 2013.

### 2.2. Statistical Analysis

SPSS 23.0 software (Chicago, IL, USA) was used for statistical processing. Continuous variables were expressed as means ± standard deviation or median (IQR), and categorical variables as frequencies. The data were then compared using Student's *t*-test, Kruskal–Wallis test, or chi-square test. Kaplan–Meier curves were used to show the cumulative incidence of HCC, and multivariate Cox regression analysis was used to analyze the risk factors for HCC development. *P* values of <0.05 were considered to be statistically significant.

## 3. Results

### 3.1. Baseline Characteristics of Patients with HBV-Related DC

**Figure 1:**
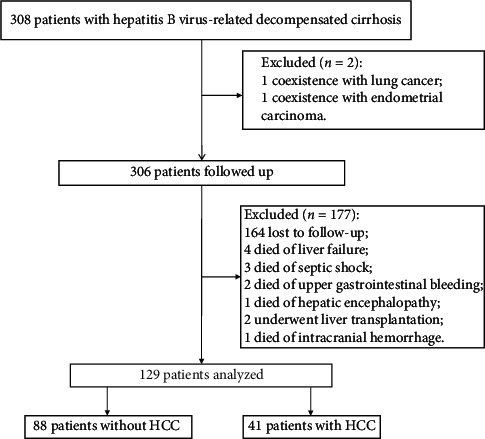
Screening of patients with hepatitis B virus-related decompensated cirrhosis.

Among the 308 patients, 2 patients with malignant tumor were excluded, 11 patients died due to diseases other than HCC, 2 patients underwent liver transplantation, and 164 patients have lost to follow-up (Figure 1). Data from the remaining 129 patients with definite records were analyzed. The median follow-up time was 5 years (range, 1 to 8 years).

The baseline characteristics of 129 patients are presented in Table 1. More than half of the patients were males (78/129, 60.5%), with an average age of 53.81 years. Most of the patients (97/129, 75.2%) were HBeAg negative and had positive HBV DNA levels (80/129, 62.1%). Among the 129 patients, 72 patients received nucleosides monotherapy, 21 patients received nucleotides monotherapy, 15 patients received combination therapy with nucleosides and nucleotides, and the remaining 21 patients received sequential therapy with nucleosides and nucleotides.

Moreover, patients who were lost to follow-up had a higher model for end-stage liver disease (MELD) scores at baseline compared with patients followed up (*P* < 0.05). The proportions of patients with gastrointestinal bleeding were higher in patients who were lost to follow-up than those in patients followed up (*P* < 0.05) (Supplementary Table, available here).

### 3.2. Baseline Characteristics of Patients with HCC

At the end of the follow-up period, 41 patients developed HCC, and the cumulative incidence of HCC was 31.8%. The median time from diagnosing DC to the incidence of HCC was 4.4 years (range, 1–7 years). The incidence of HCC was higher in males (30/78, 38.5%) when compared to females (11/51, 21.6%) (*χ*^2^ = 4.059, *P* = 0.04).

HBV viral load at baseline and viral response at sixth months during antiviral therapy between HCC and no-HCC groups were compared, and the differences showed no statistical significance (*t* = −0.54, *P* = 0.56; *χ*^2^ = 0.26, *P* = 0.60).

**Figure 2:**
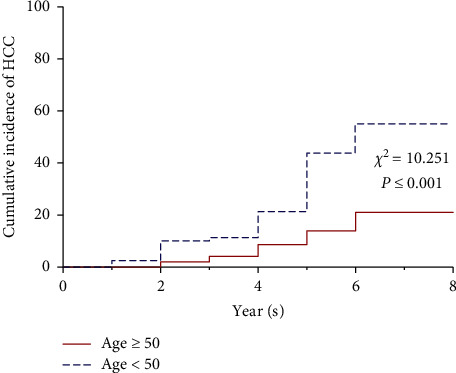
The incidence of HCC development between age <50- and ≥50-year-old groups.

Patients with developed HCC were significantly older than those who did not developed HCC (*t* = −4.482, *P* ≤ 0.001). The cutoff value of age according to MedCalc version 10.1 software for Windows (MedCalc software, Mariakerke, Belgium) was determined to be 50 years, and the incidence of HCC was compared using the Kaplan–Meier curve (*P* ≤ 0.001) (Figure 2). The results showed no significant differences in baseline platelet counts, creatinine, total bilirubin, serum sodium, AFP, and INR between the two groups (all *P* > 0.05).

Among the 60 patients with a family history of HBV infection, 15 (25.0%) developed HCC, while in 69 patients without family history, 26 (37.7%) developed HCC (*χ*^2^ = 0.639, *P* = 0.42). For 17 patients with a history of splenectomy, 5 developed HCC, while 36 of 112 patients without the history of splenectomy have developed HCC (*χ*^2^ = 0.051, *P* = 0.82).

With regard to the complications such as ascites, upper gastrointestinal bleeding, and hepatic encephalopathy, the incidence of HCC in patients with complications was similar to that in patients without complications (*χ*^2^ = 0.000, 1.907, and 0.888; *P* = 0.99, 0.27, and 0.35, respectively).

There were 129 patients who received antiviral therapy for more than a year. Of these, 72 patients received nucleoside analogues, including entecavir, lamivudine, and telbivudine, 21 patients received nucleotide analogues including adefovir and tenofovir, 15 patients received combination treatment with nucleoside and nucleotide analogues, and the remaining 21 patients received sequential therapy with nucleosides and nucleotides. The incidence of HCC in these patients was 34.7%, 38.1%, 33.3%, and 10.3%, respectively, showing no significant differences among the four groups (*χ*^2^ = 3.65, *P* = 0.29).

### 3.3. Risk Factors for HCC Development in Patients with HBV-Related DC

Univariate and multivariate Cox regression analyses were conducted to determine the risk factors of HCC development. Univariate analysis showed that male gender (*P* = 0.02) and age ≥50 years (*P* = 0.04) were found to be associated with HCC development. After that, multivariate Cox regression results revealed that male gender and age ≥50 years are independent risk factors for HCC development (OR = 2.987 and 2.408; 95% CI (1.301–6.858) and (1.126–5.149); *P* = 0.01 and 0.02, respectively) (Table 2).

## 4. Discussion

In the present study, an 8-year follow-up was conducted to investigate the incidence of HCC development in patients with DC and long-term antiviral therapy. Data showed that 41 patients developed HCC, and the cumulative incidence of HCC was 31.8%. The median time from DC diagnosis to HCC incidence was 4.4 years. Multivariate Cox regression showed that male gender and age ≥50 years are independent risk factors for HCC development.

The cumulative incidence of HCC was found to be 31.8%, which is much higher than the data reported from Korea and Europe countries [3, 4]. The main reason for the difference is that patients in the abovementioned studies have CHB or compensated LC, other than DC. Patients with HCC in the late stage have limited treatment strategies and poor prognosis, and so it is critical to detect HCC at an early stage. Patients with HBV-related DC are at high risk of HCC and should be screened for HCC in clinical practice.

Moreover, long-term antiviral therapy can result in biochemical remission and histological improvement, and even in the regression of cirrhosis, but the risk of HCC could not be eliminated, especially in patients with LC [12]. Jang et al. have reported that the incidence of HCC in patients who underwent antiviral-treated treatment and untreated DC was 32.3% and 27.2%, and antiviral therapy showed no significant effect on reducing the HCC incidence [13]. Patients with DC can benefit from maintained virologic response but had no significant reduction in the risk of HCC, or HCC-related mortality showed no significant reduction. Another study showed that no virological response to entecavir ETV was a risk factor of HCC development in patients with DC [14]. Recently, HCC development in patients who received TDF and ETV treatments has attracted much attention worldwide, but data concerning HCC development in DC were scarce. In the present study, nucleosides and nucleotides showed no significant differences in HCC development in patients with DC. As a complement to previous studies [9, 13, 15–18], this suggests that nucleotide analogues might be not superior to nucleoside analogues in reducing HCC development in patients with HBV-related DC.

Recently, data from the U.S. Cancer Statistics Registry reported that the incidence of HCC has been increased in patients older than 50 years, and males are more prone to this [19]. Similar to the previous study [19], male gender and age ≥50 years are considered as independent risk factors for HCC in patients with HBV-related DC. In addition, splenectomy showed no significant effect with regard to HCC incidence, and this is different from Lv's study [20], requiring validation in a large sample size in the future.

However, the present study had some limitations. Almost half of the patients had lost to follow-up, and this was an inevitable but true limitation in this study. Since higher baseline MELD scores and higher proportions of patients with gastrointestinal bleeding were found in patients who were lost to follow-up, it can be speculated that many patients died from DC and lost to follow-up. Another reason is that many patients were not local citizens and not convenient to return for further consultation. After all, this might not affect the conclusion of the study. Moreover, the sample size is not large, and so a multicenter, prospective study with a larger sample size is warranted.

In summary, the risk of HCC remains to be high in patients with HBV-related DC and long-term antiviral therapy, especially in male patients aged ≥50 years.

## Figures and Tables

**Table 1:** Baseline characteristics of the patients with decompensated liver cirrhosis.

	Patients with HCC (*n* = 41)	Patients without HCC (*n* = 88)	*P* value
Age, years	61 (53.5–66)	51 (44.25–58.75)	≤0.001
Age <50/age ≥50	7/34	42/46	≤0.001
Male/female	30/11	48/40	0.04
Complications, *n* (%)			
Encephalopathy	6 (14.6)	8 (9.1)	0.35
GI bleeding	1 (2.4)	8 (9.1)	0.27
Ascites	21 (51.2)	45 (51.1)	0.94
Splenectomy (*n* = 17)	5 (12.2)	12 (13.6)	0.82
Family history of CHB (Y/N)	15/26	45/43	0.40
Previous antiviral therapy			—
Nucleosides, *n* (%)	25 (61.0)	47 (53.4)	0.29
Nucleotides, *n* (%)	8 (19.5)	13 (14.8)	
Combination therapy, *n* (%)	5 (12.2)	10 (11.3)	
Sequential therapy, *n* (%)	3 (7.3)	18 (20.5)	
Laboratory findings			
HBeAg positive, *n* (%)	13 (31.7)	19 (21.6)	0.22
HBV DNA (log IU/mL)	4.17 ± 1.40	4.01 ± 1.64	0.59
HBV DNA positive at 6^th^ month, *n* (%)	7 (17.0)	12 (13.6)	0.60
ALT (U/L)	57.29 ± 55.89	52.97 ± 47.14	0.68
Total bilirubin (*μ*mol/L)	35.21 ± 18.43	35.53 ± 28.95	0.95
Cholinesterase (U/L)	4245.95 ± 1946.29	3653.83 ± 1655.46	0.12
Platelet counts (*E *+ 09/L)	71.96 ± 49.01	81.97 ± 67.06	0.40
INR	1.30 ± 0.21	1.31 ± 0.33	0.82
Sodium (mmol/L)	140.83 ± 3.20	140.45 ± 2.5	0.49
Creatinine (*μ*mol/L)	76.50 ± 17.00	75.77 ± 19.36	0.84
AFP (ng/ml)	3.28 (1.71–4.21)	4.85 (2.36–19.70)	0.14
MELD	11.63 ± 3.54	11.25 ± 4.56	0.64

Data are shown as means ± standard deviation or median (IQR). Abbreviations: HCC, hepatocellular carcinoma; GI, gastrointestinal; CHB, chronic hepatitis B; HBeAg, hepatitis B e antigen; HBV, hepatitis B virus; DNA, deoxyribonucleic acid; ALT, alanine aminotransferase; INR, prothrombin time/international normalized ratio; AFP, alpha-fetoprotein; MELD, model for end-stage liver disease.

**Table 2:** Risk factors for HCC development in patients with hepatitis B virus-related decompensated cirrhosis.

Variables	Univariate analysis		Multivariate analysis	
	OR (95% CI)	*P* value	OR (95% CI)	*P* value
Age	2.994 (1.240–7.227)	0.02	2.987 (1.301–6.858)	0.01
Sex	2.561 (1.055–6.220)	0.04	2.408 (1.126–5.149)	0.02
ALT (U/L)	0.996 (0.987–1.005)	0.35		
Total bilirubin (*μ*mol/L)	1.006 (0.987–1.026)	0.54		
Cholinesterase (U/L)	1.000 (0.999–1.000)	0.50		
Platelet counts (*E *+ 09/L)	1.000 (0.994–1.007)	0.89		
INR	0.408 (0.062–2.674)	0.35		
Sodium (mmol/L)	1.122 (0.968–1.301)	0.13		
Creatinine (*μ*mol/L)	0.998 (0.978–1.0191)	0.86		
AFP (ng/ml)	0.998 (0.998–1.002)	0.77		

Abbreviations: ALT, alanine aminotransferase; INR, prothrombin time/international normalized ratio; AFP, alpha-fetoprotein.

## Data Availability

The data used to support the findings of this study are available from the corresponding author upon request.

## Supplementary Materials

Supplementary Materials Supplementary table. Baseline characteristics of patients with decompensated Cirrhosis.
