# Supplementary material for: Risk of Hepatocellular Carcinoma Remains High in Patients with HBV-Related Decompensated Cirrhosis and Long-Term Antiviral Therapy
Source: Can J Gastroenterol Hepatol. 2020 Dec 17;2020:8871024. doi: 10.1155/2020/8871024 (PMC7762653; doi:10.1155/2020/8871024)
Supplement: Supplementary Materials — Supplementary table. Baseline characteristics of patients with decompensated Cirrhosis. [file 8871024.f1.docx]

**Supplementary table. Baseline characteristics of patients with decompensated Cirrhosis**

|  | Patients followed up  (n = 129) | Patients lost to follow-up  (n = 164) | *P* value |
| --- | --- | --- | --- |
| Age | 53.81 ± 11.12 | 53.65 ± 11.49 | 0.91 |
| Male/ female | 80/49 | 110/54 | 0.37 |
| *Complications, n* (%) |  |  |  |
| Hepatic encephalopathy | 14 (10.8) | 12 (7.3) | 0.191  0.010  0.134 |
| Gastrointestinal bleeding | 9 (6.9) | 28 (17.0) |  |
| Ascites | 66 (51.2) | 80 (48.8) |  |
| *Laboratory findings* |  |  |  |
| HBeAg positive, n (%) | 32 (24.0) | 33 (20.1) | 0.338 |
| HBV DNA (log IU/mL) | 4.06 ± 1.57 | 4.06 ± 1.59 | 0.977 |
| ALT (U/L) | 36 (24－63) | 30 (17－56) | 0.907 |
| Total bilirubin (μmol/L) | 35.42 ± 26.00 | 82.85 ± 30.62 | ≤ 0.001 |
| Platelet counts (E+09/L) | 78.79 ± 61.90 | 77.83 ± 62.24 | 0.90 |
| INR | 1.31 ± 0.30 | 1.43 ± 0.37 | 0.004 |
| Serum sodium (mmol/L) | 140.58 ± 2.80 | 137.77 ± 11.00 | 0.005 |
| Creatinine (μmol/L) | 76.53 ± 17.35 | 83.07 ± 30.42 | 0.031 |
| MELD | 11.37 ± 4.26 | 13.22 ± 4.95 | 0.001 |

Comparison was conducted by a Student’s *t*-test method (means ± standard deviation) for normal distribution conditions, a rank sum test (median and range) for non-normal distributions, and Chi-square test for categorical values. Abbreviations: HBeAg, hepatitis B e antigen; ALT, alanine aminotransferase; INR, international normalized ratio; MELD, model for end-stage liver disease.
